# A165 IL-6 ENHANCES THE ANTI-COLITIC THERAPEUTIC POTENTIAL OF HUMAN IL-4 POLARIZED MACROPHAGES

**DOI:** 10.1093/jcag/gwae059.165

**Published:** 2025-02-10

**Authors:** N Andonian, B E Callejas, J Sousa, A Wang, D McKay

**Affiliations:** University of Calgary Cumming School of Medicine, Calgary, AB, Canada; University of Calgary Cumming School of Medicine, Calgary, AB, Canada; University of Calgary Cumming School of Medicine, Calgary, AB, Canada; University of Calgary Cumming School of Medicine, Calgary, AB, Canada; University of Calgary Cumming School of Medicine, Calgary, AB, Canada

## Abstract

**Background:**

Inflammatory bowel disease (IBD) is a group of inflammatory disorders, including Crohn’s disease and ulcerative colitis, that affect the gastrointestinal tract and can significantly impact patients’ quality of life. Forty percent of individuals with IBD are unresponsive to current therapies and a cure remains elusive. Macrophages play key roles in inflammation and tissue repair, with IL-4 treated macrophages, M(IL4)s, exerting an anti-inflammatory effect. We showed that systemic administration of human M(IL4)s reduced disease severity in murine colitis. Interleukin-6 is considered a pro-inflammatory cytokine, the levels of which are increased in IBD gut tissue. Murine studies suggest IL-6 may reinforce an M(IL4) phenotype, raising the possibility, and perhaps challenging dogma, that IL-6 could promote the beneficial effects of M(IL4)s. Therefore, I *hypothesize* that local tissue signal IL-6 enhances the anti-inflammatory properties of human M(IL4)s.

**Aims:**

1. Determine the effect of IL-6 on the phenotype of human M(IL4)s.

2. Determine the effect of IL-6 on M(IL4) function, assessing (a) wound healing capacity and (b) cell death.

3. Compare the efficacy of M(IL4) and M(IL4+IL6) in the DNBS-*rag1*^-/-^ mouse colitis.

**Methods:**

M(IL4)s were differentiated from blood monocytes from male and female healthy volunteers and individuals with Crohn’s disease. M(IL4)s ± IL-6 (10 ng/mL; 24h) were assessed for (1) phenotype (mRNA expression) and (2) function. Function was evaluated through testing (a) conditioned medium from M(IL4+IL6)s in an *in vitro* Caco2 epithelial scratch-wound assay and (b) viability in response to hydrogen peroxide (H_2_O_2_) (oxidative stress) via LDH release. (3) M(IL4)s or M(IL4+IL6)s were delivered intraperitoneally (10^6^ cells) and 48h later, *rag1*^*-/-*^ mice received 5mg intra-rectal DNBS. Colitis was assessed 72h later. Non-treated M(0) and M(IL6) serve as controls.

**Results:**

M(IL4)s and M(IL4+IL6)s show similar increased expression of CD206 and CCL18, and decreased CD14 mRNA (n=6-7). Conditioned media from both M(IL4)s and M(IL4+IL6)s promoted a similar degree of epithelial migration in the wound healing assay (n=5). Macrophages from patients had increased susceptibility to H_2_O_2_-induced death, which was reduced in the M(IL4+IL6) group (n=6 healthy donors; n=6 Crohn’s). M(IL4+IL6)s were more effective than M(IL4)s in protecting against murine colitis as determined by body weight, colon length and a macroscopic disease score (n=8 mice, 3 exps.).

**Conclusions:**

IL-6 does not abrogate the M(IL4) phenotype or wound healing capacity and protects the cell from H_2_O_2_-induced cytotoxicity. Also, M(IL4+IL6)s have a superior anti-colitic effect when compared to M(IL4)s from the same donor. The data support macrophage transfer to treat colitis and indicate that an element of an inflamed environment can enhance the anti-colitic effect of human M(IL4)s.

**Funding Agencies:**

CIHRCartier Family Student Research Award in Gastroenterology and Hepatology, Alberta Graduate Excellence Scholarship, Helmsley Grant

